# CDDO-Me Selectively Attenuates CA1 Neuronal Death Induced by Status Epilepticus via Facilitating Mitochondrial Fission Independent of LONP1

**DOI:** 10.3390/cells8080833

**Published:** 2019-08-05

**Authors:** Ji-Eun Kim, Hana Park, Seo-Hyeon Choi, Min-Jeong Kong, Tae-Cheon Kang

**Affiliations:** 1 Department of Anatomy and Neurobiology, College of Medicine, Hallym University, Chuncheon 24252, Korea; 2 Institute of Epilepsy Research, College of Medicine, Hallym University, Chuncheon 24252, Korea

**Keywords:** 4-HNE, DRP1, ERK1/2, hippocampus, JNK, mitochondrial dynamics, PKA, protein phosphatases, TUNEL

## Abstract

2-Cyano-3,12-dioxo-oleana-1,9(11)-dien-28-oic acid methyl ester (CDDO-Me) is a triterpenoid analogue of oleanolic acid that exhibits promising anti-cancer, anti-inflammatory, antioxidant and neuroprotective activities. In addition, CDDO-Me affects cellular differentiation and cell cycle arrest, and irreversibly inhibits Lon protease-1 (LONP1). In the present study, we evaluate the effects of CDDO-Me on mitochondrial dynamics and its downstream effectors in order to understand the underlying mechanism of the neuronal death following status epilepticus (SE, a prolonged seizure activity). CDDO-Me increased dynamin-related proteins 1 (DRP1)-serine 616 phosphorylation via activating extracellular-signal-regulated kinase 1/2 (ERK1/2) and c-Jun *N*-terminal kinase (JNK), but not protein kinase A (PKA) or protein phosphatases (PPs). In addition, CDDO-Me facilitated DRP1-mediated mitochondrial fissions, which selectively attenuated SE-induced CA1 neuronal death. Unlike CDDO-Me, LONP1 knockdown led to SE-induced massive degeneration of dentate granule cells, CA1 neurons and hilus interneurons without altering the expression and phosphorylation of DRP1, ERK1/2, JNK and PP2B. LONP1 knockdown could not inhibit SE-induced mitochondrial elongation in CA1 neurons. Co-treatment of CDDO-Me with LONP1 siRNA ameliorated only CA1 neuronal death, concomitant with abrogation of mitochondrial elongation induced by SE. Thus, our findings suggest that CDDO-Me may selectively attenuate SE-induced CA1 neuronal death by rescuing the abnormal mitochondrial machinery, independent of LONP1 activity.

## 1. Introduction

Status epilepticus (SE) is a condition which shows prolonged and uncontrolled seizure activity [1]. One of the most remarkable SE-induced consequences is a massive neuronal death, which triggers long-term and profound alterations in the neuronal network that lead to the development of temporal lobe epilepsy (TLE) [2,3,4]. The neuronal death pattern and susceptibility to SE shows the regional specific heterogeneity: Neurons in the hilus region of the dentate gyrus, such as mossy cells and hilus interneurons (in particular parvalbumin (PV) interneurons), are the most vulnerable to SE insults, while dentate granule cells are less vulnerable. Furthermore, SE induces programmed necrotic CA1 neuronal death, while it evokes apoptosis in the hilus region [5,6,7,8,9,10]. Therefore, information regarding the molecular events underlying neuron-specific vulnerability may be one of the therapeutic strategies for neuroprotection against SE, which inhibits undesirable output including epileptogenesis.

Mitochondria are dynamic organelles of eukaryotic cells responsible for generating ATP. In addition, mitochondria participate in the synthesis of reactive oxygen species (ROS), cell homeostasis and calcium buffering. In the process of supplying ATP, mitochondria produce ROS through the electron transport chain, which induces oxidative stress. ROS production and oxidative damage induced by neuronal insults rapidly change mitochondrial morphologies, although mitochondria-derived ROS act as homeostatic signaling molecules in various physiological processes [11,12,13,14,15]. To exert their functions properly, mitochondria change their morphologies by two continuous antagonistic processes: fusion and fission (mitochondrial dynamics) [11]. As vital determinants of the fission-fusion balance, various proteins share reciprocal relationships [12]. Briefly, mitochondrial fusion (elongation) is regulated by mitofusin 1/2 (MFN1/2) and optic atrophy 1 (OPA1), while fission (fragmentation) is modulated by dynamin-related proteins 1 (DRP1) [11,12,13]. Thus, perturbation of mitochondrial dynamics and the altered expressions/activities of regulatory enzymes lead to neurodegeneration. In particular, the post-translational modification of DRP1 is focused on various neurodegenerative disorders. DRP1 activity is reversely regulated by phosphorylation of serine (S) 616 and 637 sites: DRP1-S616 phosphorylation facilitates mitochondrial fission. However, DRP1-S637 phosphorylation leads to DRP1 detaching from mitochondria, which subsequently inhibits mitochondrial fission. *S*-nitrosylation of cysteine 644 by nitric oxide (NO) also facilitates mitochondrial fragmentation by increasing S616 phosphorylation. Thus, the imbalance of DRP1 phosphorylation and *S*-nitrosylation evokes dysfunctions of mitochondrial dynamics, which are involved in pathological processes such as cancer and neurological diseases [14,15].

Interestingly, the impaired mitochondrial dynamics distinctly participate in heterogeneous neuronal death patterns in the hippocampus. Briefly, PV interneurons in the hilus regions show apoptotic degeneration following SE, accompanied by extensive mitochondrial fission [10]. In contrast, the abnormal elongation of swollen mitochondria contributes to CA1 neuronal necrosis, which is initiated by aberrant cell cycle entry in post-mitotic neurons [7,8,16,17,18]. Given these previous reports, the stoichiometric relationship between fission and fusion plays an important role in neuronal viability. Thus, insight into the molecular mechanism responsible for the impaired mitochondrial dynamics provides a deeper understanding of the distinct vulnerability of a neuronal subpopulation to SE.

2-Cyano-3,12-dioxo-oleana-1,9(11)-dien-28-oic acid methyl ester (CDDO-Me) is a triterpenoid analogue of oleanolic acid that exhibits promising anti-cancer, anti-inflammatory, antioxidant and neuroprotective activities. CDDO-Me affects cellular differentiation and cell cycle arrest [19,20]. CDDO-Me also irreversibly inhibits Lon protease-1 (LONP1) activity by forming covalent LONP1-CDDO adducts [21,22]. LONP1 is a highly conserved serine peptidase that contributes to protein quality control processes [23], which play a crucial role in maintaining mitochondrial morphology and function [24]. With respect to these previous studies, exploring the effects of CDDO-Me on mitochondrial dynamics is noteworthy in order to understand the mechanism of the distinct neuronal death that occurs in response to SE, which has been elusive. 

Here, we demonstrate previously unsuspected effects of CDDO-Me on DRP1-mediated mitochondrial fissions, which selectively attenuated SE-induced CA1 neuronal death via activating extracellular-signal-regulated kinase 1/2 (ERK1/2) and c-Jun *N*-terminal kinase (JNK). In contrast to CDDO-Me, LONP1 knockdown aggravated SE-induced neuronal death without changing ERK1/2 and JNK activities. Co-treatment of CDDO-Me with LONP1 siRNA ameliorated only CA1 neuronal death, concomitant with abrogation of mitochondrial elongation induced by SE. Thus, our findings suggest CDDO-Me may abrogate abnormal mitochondrial machinery-mediated neuronal death induced by SE, independent of LONP1 activity.

## 2. Materials and Methods

### 2.1. Experimental Animals and Chemicals

Male Sprague–Dawley (SD) rats (7 weeks old, Daehan Biolink, South Korea) were used in the present study. Animals were given a commercial diet and water ad libitum under controlled conditions (22 ± 2 °C, 55 ± 5% and a 12:12 light/dark cycle with lights). Animal protocols were approved by the Institutional Animal Care and Use Committee of Hallym University (Chuncheon, Republic of Korea). The number of animals used and their suffering were minimized in all cases. All reagents were obtained from Sigma-Aldrich (St. Louis, MO, USA), except as noted.

### 2.2. Surgery, CDDO-Me Infusion and LONP1 Knockdown

Under Isoflurane anesthesia (3% induction, 1.5–2% for surgery and 1.5% maintenance in a 65:35 mixture of N_2_O:O_2_), animals were infused with each chemical or siRNA into the right lateral ventricle (1 mm posterior; 1.5 mm lateral; −3.5 mm depth to the bregma) with a brain infusion kit 1 and an Alzet 1007D osmotic pump (Alzet, Cupertino, CA, USA). The osmotic pump contained (1) vehicle, (2) CDDO-Me (10 μM), (3) a non-targeting control siRNA (5-GCAACUAACUUCGUUAGAAUCGUUAUU-3), (4) LONP1 siRNA (5-GAGACAAGUUGCGCAUGAUTT-3) and (5) CDDO-Me + LONP1 siRNA. The pump was placed in a subcutaneous pocket in the dorsal region. Some animals were also implanted with a monopolar stainless steel electrode (Plastics One, USA) into the left dorsal hippocampus (−3.8 mm posterior; 2.0 mm lateral; −2.6 mm depth). The connecting wire and electrode socket were then inserted in an electrode pedestal (Plastics One, USA) and secured to the exposed skull with dental acrylic [8,16,17,18]. Three days after surgery, rats were induced with SE by lithium chloride (LiCl)-pilocarpine.

### 2.3. SE Induction and Electroencephalogram (EEG) Analysis

SE was induced by a single dose (30 mg/kg) of pilocarpine in rats pretreated (24 h before pilocarpine injection) with 127 mg/kg LiCl, as previously described [8,16,17,18]. Before pilocarpine injection, animals were given atropine methylbromide (5 mg/kg i.p.) to block the peripheral effect of pilocarpine. Two hours after SE, animals received diazepam (10 mg/kg, i.p.) to terminate SE. As controls, rats were treated with saline instead of pilocarpine. For evaluation of the effects of CDDO-Me infusion and LONP1 siRNA knockdown on seizure susceptibility in response to pilocarpine, some animals’ EEG signals were recorded with a DAM 80 differential amplifier (0.1–3000 Hz bandpass; World Precision Instruments, Sarasota, FL, USA). EEG activity was measured during the 2 h recording session from each animal. The data were digitized (400 Hz) and analyzed using LabChart Pro v7 (AD Instruments, New South Wales, Australia). Time of seizure onset was defined as the time point showing paroxysmal depolarizing shift, which lasted more than 3 seconds and consisted of a rhythmic discharge between 4 and 10 Hz with an amplitude of at least two times higher than the baseline EEG. EEG activity was measured during the 2 h recording session from each animal. Spectrograms were also automatically calculated using a Hanning sliding window with 50% overlap. Two hours after SE onset, diazepam (Valium; Roche, France; 10 mg/kg, i.p.) was administered and repeated, as needed [18].

### 2.4. Tissue Processing

Three days after SE induction, animals were perfused transcardially with phosphate-buffered saline (PBS, pH 7.4) followed by 4% paraformaldehyde in 0.1 M phosphate buffer (PB, pH 7.4) under urethane anesthesia (1.5 g/kg i.p.). The brains were removed, postfixed in the same fixative for 4 h and rinsed in PB containing 30% sucrose at 4 °C for 2 days. Thereafter, the tissues were frozen and sectioned with a cryostat at 30 μm, and consecutive sections were collected in six-well plates containing PBS. For western blot, animals were decapitated under urethane anesthesia. The hippocampus was rapidly removed and homogenized in lysis buffer. The protein concentration in the supernatant was determined using a Micro BCA Protein Assay Kit (Pierce Chemical, USA).

### 2.5. Immunohistochemistry and Measurements of Mitochondrial Length and Neuronal Damage

After incubation with 10% normal goat serum (Vector, Burlingame, CA, USA), sections were incubated in a mixture of primary antibodies shown in Table 1 (in PBS containing 0.3% triton X-100) at room temperature, overnight. After washing, sections were incubated for 1 h in a fluorescein isothiocyanate (FITC, green)-, Cy3 (red)- or aminomethylcoumarin acetate (AMCA, blue)-conjugated secondary antibodies (Vector, Burlingame, CA, USA). For negative control, tissues were incubated in pre-immune serum instead of primary antibody. Negative control tissues did not show any immunoreactivity for the primary antibody (data not shown). Images were captured using an AxioImage M2 microscope or a confocal laser scanning microscope (LSM 710, Carl Zeiss Inc., Oberkocken, Germany). Individual mitochondrion length in PV cells and CA1 neurons (*n* = 20/section) was measured using ZEN lite software (Blue Edition, Carl Zeiss Inc., Oberkocken, Germany) following 3D-reconstruction. Based on our previous study [10,18], twenty-five serial images (z-stack, 1 μm) were obtained from each hippocampal section. Serial images were stacked, aligned, visualized and converted to 3D images using the ZEN lite program. Thereafter, individual mitochondrial length (long axis) was measured. In addition, Fluoro-Jade B (FJB) and terminal deoxynucleotidyl transferase dUTP nick end labeling (TUNEL) staining were performed according to the manufacturer’s instructions to analyze the neuronal damage. Two different investigators who were blind to the classification of tissues performed the measurement of mitochondrial length and the cell count of FJB and TUNEL positive neurons based on the lamellar structure of the hippocampus. For quantitative analysis of fluorescent intensity, sections (15 sections per each animal) were viewed through a microscope connected via Axiocam camera (Carl Zeiss Korea, Seoul, South Korea). Thereafter, fluorescent intensity measurements were represented as the number of a 256-gray scale using AxioVision Rel. 4.8 software (Carl Zeiss Korea, Seoul, South Korea). Intensity values were corrected by subtracting the average values of background noise obtained from five image inputs. The optical density was then standardized by setting the threshold levels.

### 2.6. Western Blot

Western blot was performed by the standard protocol. The primary antibodies used in the present study are listed in Table 1. The bands were detected and quantified on ImageQuant LAS4000 system (GE Healthcare, USA). As an internal reference, rabbit anti-β-actin primary antibody (1:5000) was used. The values of each sample were normalized with the corresponding amount of β-actin. The ratio of phosphoprotein to total protein was described as the phosphorylation level.

### 2.7. Quantification of Data and Statistical Analysis

All data were analyzed using the Mann-Whitney test or ANOVA to determine statistical significance. Bonferroni’s test was used for post hoc comparisons. A *p*-value below 0.05 was considered statistically significant.

## 3. Results

### 3.1. CDDO-Me Distinctly Affects SE-Induced Neuronal Death in the Hippocampus

**Figure 1:**
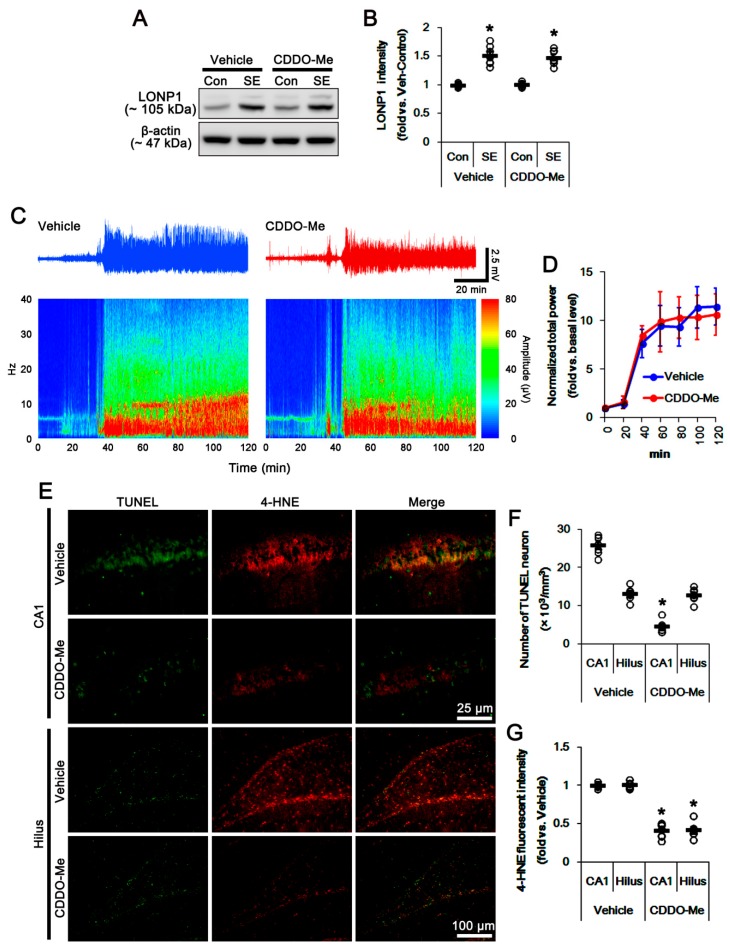
Effects of 2-Cyano-3,12-dioxo-oleana-1,9(11)-dien-28-oic acid methyl ester (CDDO-Me) on Lon protease-1 (LONP1) expression, seizure activity and neuronal damage in response to pilocarpine. (**A**–**B**) Effect of CDDO-Me on LONP1 expression in response to pilocarpine. Pilocarpine-induced status epilepticus (SE) increases LONP1 expression, which is not affected by CDDO-Me. (**A**) Representative western blots of LONP1 expression. (**B**) Quantification of LONP1 expression based on western blot data. Open circles indicate each individual value. Horizontal bars indicate mean value (mean ± S.E.M.; * *p* < 0.05 vs. control animals, respectively; *n* = 7). (**C**–**D**) Effect of CDDO-Me on seizure activity in response to pilocarpine. CDDO-Me does not influence seizure activity induced by pilocarpine. (**C**) Representative electroencephalogram (EEG) traces and frequency-power spectral temporal maps in response to pilocarpine. (**D**) Quantification of total EEG power (seizure intensity) in response to pilocarpine. Open circles indicate each individual value. Horizontal bars indicate mean value (mean ± S.E.M.; *n* = 7, respectively). (**E**–**G**) Effects of CDDO-Me on neuronal death and 4-hydroxy-2-nonenal (4-HNE) signals following SE. CDDO-Me mitigates CA1 neuronal damage, but not hilus interneurons, although it reduces 4-HNE signals in both neurons. (**E**) Representative photos of double immunofluorescent staining for terminal deoxynucleotidyl transferase dUTP nick end labeling (TUNEL) and 4-HNE. (**F**) Quantifications of the number of TUNEL positive neurons and (**G**) and the fluorescent intensity of 4-HNE in response to pilocarpine. Open circles indicate each individual value. Horizontal bars indicate mean value (mean ± S.E.M.; * *p* < 0.05 vs. vehicle; *n* = 7, respectively).

Figure 1 shows that SE resulted in up-regulation of LONP1 to ~1.5-fold of the control level in the whole hippocampus, accompanied by massive neuronal death in the CA1 region and the hilus of the dentate gyrus (*p* < 0.05 vs. control animals, respectively; Figure 1A,B,E,F). CDDO-Me did not affect the seizure latency and its severity in response to pilocarpine (Figure 1C,D). Consistent with a previous report [22], CDDO-Me did not affect LONP1 protein levels in the whole hippocampus of control- and post-SE animals, as compared to vehicle (Figure 1A,B). CDDO-Me effectively attenuated SE-induced neuronal loss in the CA1 region, but not in the hilus region (*p* < 0.05 vs. vehicle; Figure 1E–F), although it abolished 4-hydroxy-2-nonenal (4-HNE, the end-product of lipid peroxidation) signals in both regions (*p* < 0.05 vs. vehicle; Figure 1E,G). These findings indicate that CDDO-Me may differently affect the regional specific neuronal death following SE, independent of ROS generation.

### 3.2. CDDO-Me Induces Mitochondrial Fission in CA1 Neuron and PV Cells without Altering LONP1 Expression

**Figure 2:**
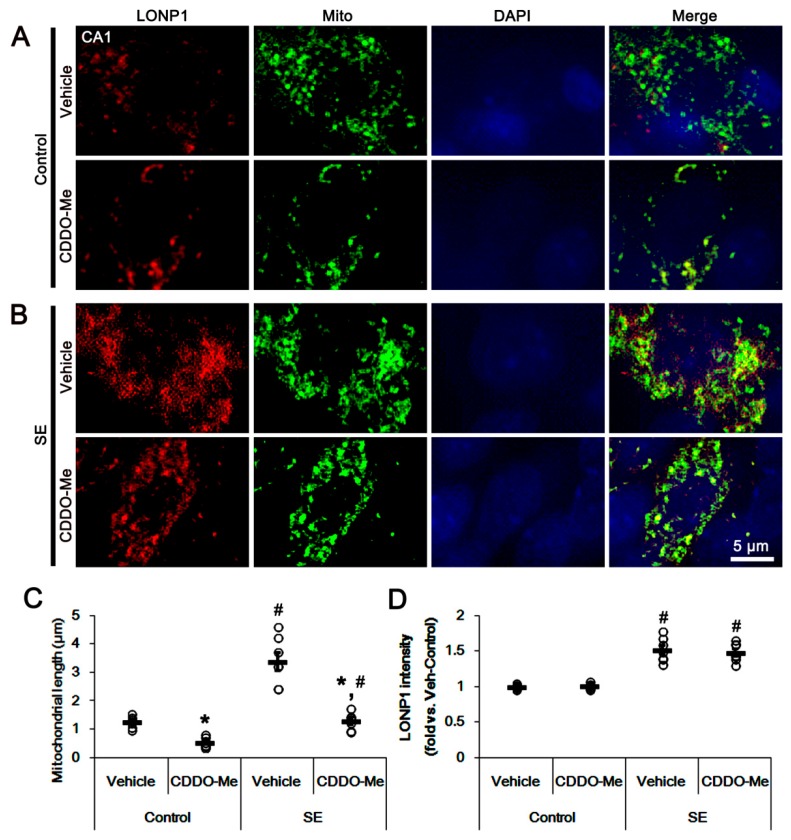
Effects of CDDO-Me on mitochondrial length and LONP1 expression in CA1 neurons. CDDO-Me reduces mitochondrial length in CA1 neurons of (**A**) control- and (**B**) post-SE animals. CDDO-Me does not affect the increased LONP1 expression following SE. (**A**,**B**) Representative photos of LONP1, mitochondria (Mito) and nuclei (DAPI) in CA1 neurons. (**C**,**D**) Quantifications of (**C**) the mitochondrial length and (**D**) LONP1 fluorescent intensity in CA1 neurons. Open circles indicate each individual value. Horizontal bars indicate mean value. Error bars indicate SEM (*^,#^ *p* < 0.05 vs. vehicle and control animals, respectively; *n* = 7, respectively).

**Figure 3:**
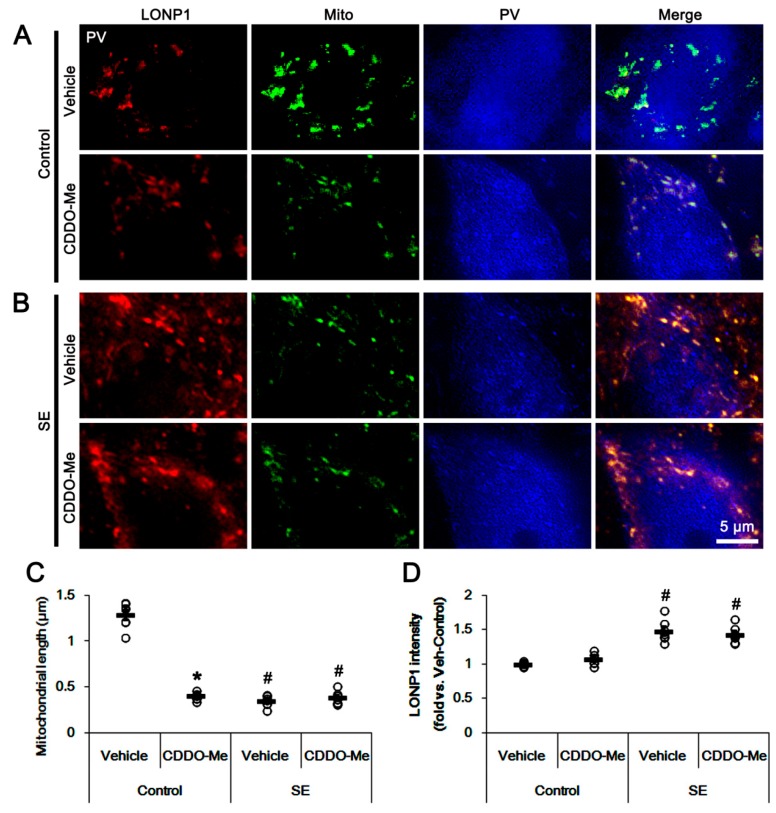
Effects of CDDO-Me on mitochondrial length and LONP1 expression in parvalbumin (PV) cells. CDDO-Me reduces mitochondrial length in CA1 neurons of (**A**) control- and (**B**) post-SE animals. CDDO-Me does not affect the increased LONP1 expression following SE. (**A**,**B**) Representative photos of LONP1, mitochondria (Mito) and PV. (**C**,**D**) Quantifications of (**C**) the mitochondrial length and (**D**) LONP1 fluorescent intensity in PV cells. Open circles indicate each individual value. Horizontal bars indicate mean value. Error bars indicate SEM (*^,#^ *p* < 0.05 vs. vehicle and control animals, respectively; *n* = 7, respectively).

As mentioned previously, the dysfunctions of mitochondrial dynamics induced by SE lead to apoptosis and programmed necrosis in PV cells and CA1 neurons, respectively [7,8,10,16,17,18]. Thus, we evaluated the effects of CDDO-Me on mitochondrial dynamics in control- and post-SE animals. In control animals, CDDO-Me reduced mitochondrial length in CA1 neurons without altering mitochondrial LONP1 expression (*p* < 0.05 vs. vehicle; Figure 2A–D). Following SE, CDDO-Me significantly inhibited mitochondrial elongation, but not mitochondrial LONP1 over-expression, in these neurons (*p* < 0.05 vs. control animals; Figure 2A–D). Similarly, CDDO-Me led to mitochondrial fragmentation in PV cells in control animals without changing mitochondrial LONP1 expression (*p* < 0.05 vs. vehicle; Figure 3A–D), while it could not affect SE-induced excessive mitochondrial fission and LONP1 over-expression (Figure 3A–D). These findings indicate that CDDO-Me may facilitate mitochondrial fission in CA1 neurons as well as PV cells, which reversely influences neuronal death in both neuronal subpopulations following SE [10,25].

### 3.3. CDDO-Me Enhances DRP1-S616 Phosphorylation in Control- and Post-SE Animals

**Figure 4:**
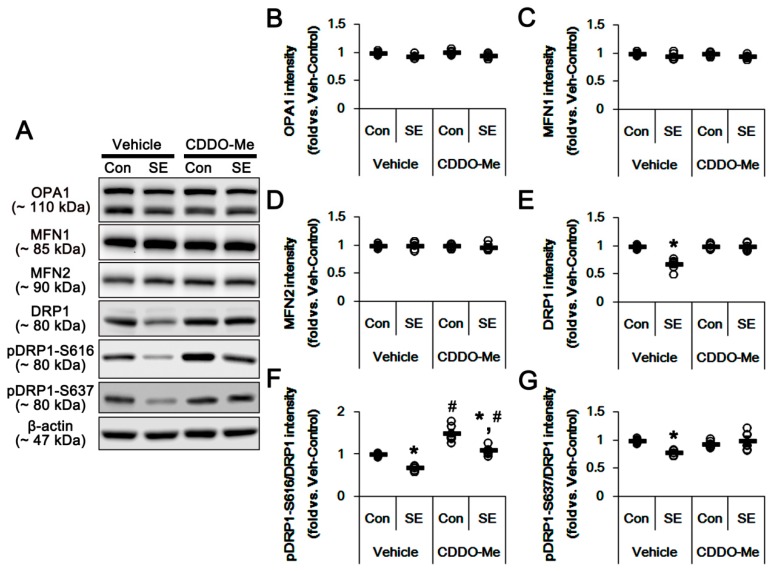
Effects of CDDO-Me on the expression and phosphorylation of mitochondrial dynamics-related molecules. CDDO-Me increases only the dynamin-related proteins 1 (DRP1)-S616 phosphorylation level under physiological- and post-SE conditions. (**A**) Representative images for western blots of optic atrophy 1 (OPA1), mitofusin 1 (MFN1), MFN2, DRP1, phospho (p)-DRP1-S616 and pDRP1-637 in the hippocampal tissues. (**B**–**G**) Quantifications of OPA1, MFN1 and MFN2, DRP1, pDRP1-S616 and pDRP1-637 levels. Open circles indicate each individual value. Horizontal bars indicate mean value. Error bars indicate SEM (*^,#^ *p* < 0.05 vs. control animals and vehicle, respectively; *n* = 7, respectively).

Since CDDO-Me exerted mitochondrial fragmentation in control and post-SE animals, we explored whether CDDO-Me influences the machinery molecules of mitochondrial dynamics. SE did not affect OPA1 and MFN1/2 expression levels (Figure 4A–D). Consistent with our previous studies [8,10,16,17,18,25], DRP1 expression was reduced to 0.68-fold of the control level following SE (*p* < 0.05 vs. control animals; Figure 4A,E). Furthermore, SE decreased the DRP1-S616 phosphorylation level to 0.67-fold of the control level (*p* < 0.05 vs. control animals; Figure 4A,F), while it did not affect the DRP1-S637 phosphorylation level (Figure 4A,G). These findings indicate a reduction in the DRP1-S616/S637 phosphorylation ratio following SE. In control animals, CDDO-Me did not influence OPA1, MFN1/2 and DRP1 expression levels (Figure 4A–E). However, CDDO-Me increased the DRP1-S616, but not S637, phosphorylation level to 1.5-fold of the vehicle level (*p* < 0.05 vs. vehicle; Figure 4A,F,G). In addition, CDDO-Me attenuated the down-regulation of DRP1 expression and its S616 phosphorylation level induced by SE. (*p* < 0.05 vs. control animals and vehicle, respectively; Figure 4A,F,G). Therefore, CDDO-Me increased the DRP1-S616/S637 phosphorylation ratio under physiological- and post-SE conditions. Since S616 site phosphorylation of DRP1 facilitates mitochondrial fission [26], our findings suggest that CDDO-Me may result in mitochondrial fragmentation via the enhancement of DRP1-S616 phosphorylation. 

### 3.4. CDDO-Me Increases ERK1/2 and JNK Activities in the Hippocampus

**Figure 5:**
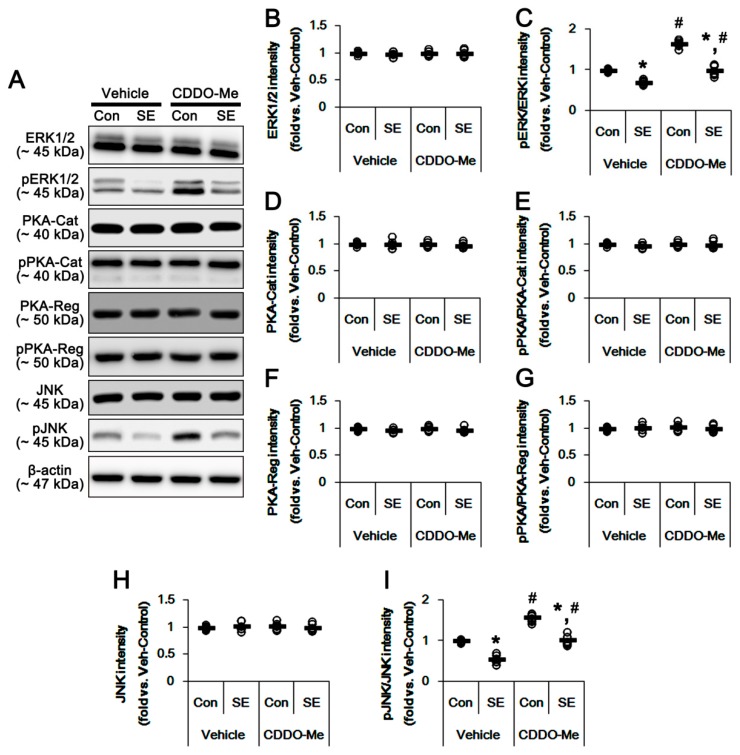
Effects of CDDO-Me on expressions and phosphorylations of extracellular-signal-regulated kinase 1/2 (ERK1/2), protein kinase A (PKA) and c-Jun N-terminal kinase (JNK). CDDO-Me increases ERK1/2 and JNK phosphorylation levels, but not PKA catalytic (PKA-Cat) and regulatory (PKA-Reg) subunits under physiological- and post-SE conditions. (**A**) Representative images for western blot of ERK1/2, phospho (p)-ERK1/2, PKA, pPKA, JNK and pJNK in the hippocampal tissues. (**B**–**G**) Quantifications of ERK1/2, pERK1/2, PKA, pPKA, JNK and pJNK levels. Open circles indicate each individual value. Horizontal bars indicate mean value. Error bars indicate SEM (*^,#^ *p* < 0.05 vs. control animals and vehicle, respectively; *n* = 7, respectively).

DRP1 phosphorylations are modulated by the ERK1/2, PKA and JNK signal pathways [26,27,28,29,30]. Furthermore, CDDO-Me influences ERK1/2 [20,31] and JNK phosphorylations [32,33]. Therefore, we evaluated the effects of CDDO-Me on the activities (phosphorylations) of protein kinases that are involved in DRP1 phosphorylations. In the present study, SE significantly reduced ERK1/2 and JNK phosphorylation levels without changing their expression (*p* < 0.05 vs. control animals, Figure 5A,B,C,F,G), while it did not affect the expression and phosphorylation levels of PKA catalytic and regulatory subunits (Figure 5A,D,E). In control animals, CDDO-Me did not change the protein expression levels of ERK1/2, PKA and JNK (Figure 5A,B,D,F). However, CDDO-Me increased the phosphorylation levels of ERK1/2 and JNK, but not PKA catalytic and regulatory subunits (*p* < 0.05 vs. vehicle; Figure 5A,C,G). Furthermore, CDDO-Me mitigated SE-induced reductions in ERK1/2 and JNK phosphorylations (*p* < 0.05 vs. vehicle; Figure 5A,C,G).

**Figure 6:**
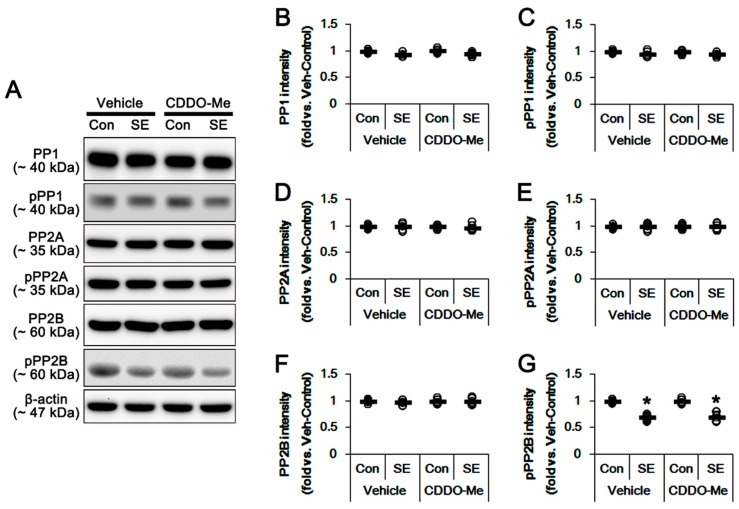
Effects of CDDO-Me on the expression and phosphorylation of protein phosphatase (PP) 1, PP2A and PP2B. CDDO-Me does not affect the expression and phosphorylation levels of protein phosphatase 1 (PP1), PP2A and PP2B under physiological- and post-SE conditions. (**A**) Representative images for western blot of PP1, phospho (p)-PP1, PP2A, pPP2A, PP2B and pPP2B in the hippocampal tissues. (**B**–**G**) Quantifications of PP1, pPP1, PP2A, pPP2A, PP2B and pPP2B levels. Open circles indicate each individual value. Horizontal bars indicate mean value. Error bars indicate S.E.M. (* *p* < 0.05 vs. control animals; *n* = 7, respectively).

Protein phosphatases also regulate mitochondrial dynamics via DRP1 dephosphorylations. Indeed, protein phosphatase (PP) 2B (calcineurin) facilitates mitochondrial fission by dephosphorylating DRP1-S637 [34]. Thus, we validated whether CDDO-Me affects PP activities in the hippocampus. In control animals, CDDO-Me did not alter the protein expressions of PP1, PP2A and PP2B and their phosphorylation levels (Figure 6A–G). SE significantly reduced PP2B (not PP1 and PP2A) phosphorylation levels in the hippocampus, indicating an increase in its activity (*p* < 0.05 vs. control animals, Figure 6A,C,E,G), which was not affected by CDDO-Me (Figure 6A,C,E,G). Taken together, our findings indicate that CDDO-Me may regulate DRP1 S616 phosphorylation by enhancing ERK1/2 and JNK activities, independent of PP activities.

### 3.5. Effects of CDDO-Me on SE-Induced Neuronal Death and Mitochondrial Dynamics are Independent of LONP1 Activity

**Figure 7:**
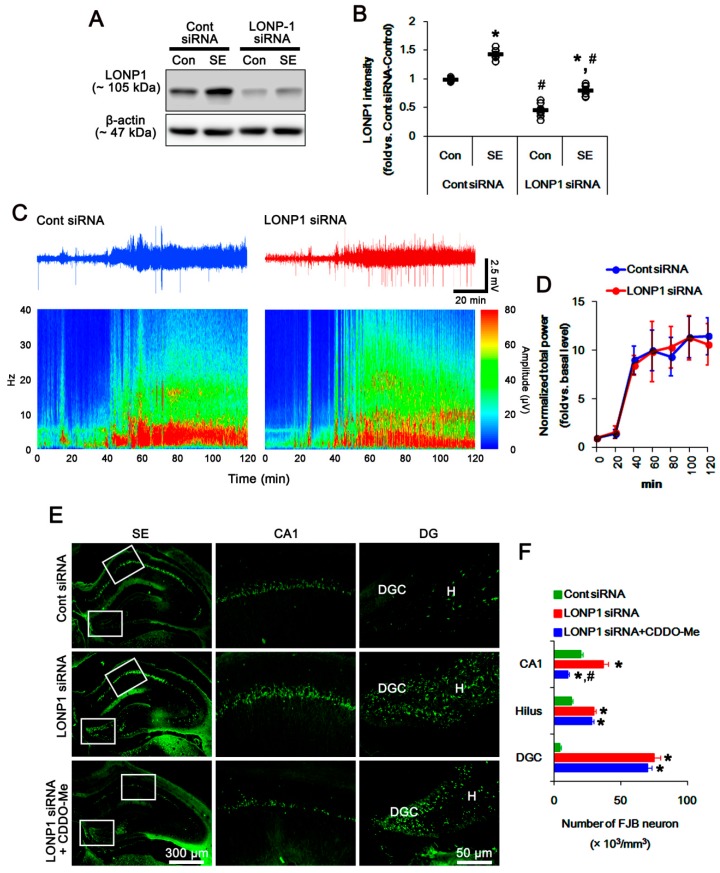
Effects of LONP1 siRNA on LONP1 expression, seizure activity and neuronal damage in response to pilocarpine. (**A**,**B**) Effect of LONP1 knockdown on LONP1 expression in response to pilocarpine. LONP1 siRNA effectively reduced LONP1 expression under physiological- and post-SE conditions. (**A**) Representative western blots of LONP1 expression. (**B**) Quantification of LONP1 expression based on western blot data. Open circles indicate each individual value. Horizontal bars indicate mean value (mean ± S.E.M.; * *p* < 0.05 vs. control animals, respectively; *n* = 7). (**C**,**D**) Effect of LONP1 knockdown on seizure activity in response to pilocarpine. LONP1 siRNA does not influence seizure activity induced by pilocarpine. (**C**) Representative EEG traces and frequency-power spectral temporal maps in response to pilocarpine. (**D**) Quantification of total EEG power (seizure intensity) in response to pilocarpine. Open circles indicate each individual value. Horizontal bars indicate mean value (mean ± S.E.M.; *n* = 7, respectively). (**E**,**F**) Effects of LONP1 siRNA and co-treatment of CDDO-Me on neuronal death induced by pilocarpine. LONP1 knockdown deteriorates degenerations of CA1 neurons, hilus interneurons (**H**) and dentate granule cells (DGC). Co-treatment of CDDO-Me attenuated CA1 neuronal death induced by SE. (**E**) Representative photos of FJB positive degenerating neurons. (**F**) Quantification of the number of FJB positive neurons in response to pilocarpine (mean ± S.E.M.; *^,#^ *p* < 0.05 vs. control siRNA and LONP1 siRNA, respectively; *n* = 7, respectively).

**Figure 8:**
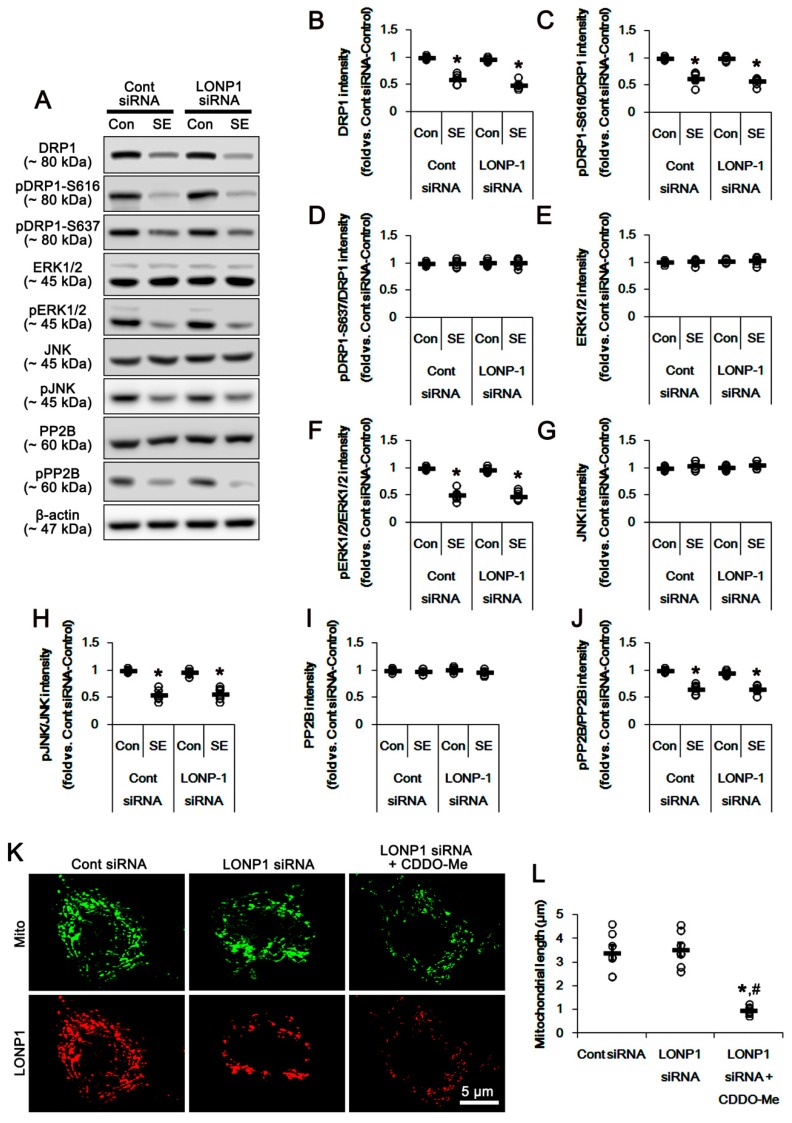
Effects of LONP1 knockdown on expression/phosphorylation of DRP1, ERK1/2, JNK and PP2B, and mitochondrial length in CA1 neurons. LONP1 siRNA does not affect the expression and phosphorylation levels of DRP1, ERK1/2, JNK and PP2B under physiological- and post-SE conditions. In addition, LONP1 knockdown does not influence SE-induced mitochondrial elongation in CA1 neurons, although it reduces LONP1 expression. However, co-treatment of CDDO-Me ameliorates mitochondrial elongation. (**A**) Representative images for western blots of DRP1, phospho (p)-DRP1-S616, pDRP1-637, ERK1/2, pERK1/2, JNK, pJNK, PP2B and pPP2B in the hippocampal tissues. (**B**–**J**) Quantifications of DRP1, pDRP1-S616, pDRP1-637, ERK1/2, pERK1/2, JNK, pJNK, PP2B and pPP2B levels. Open circles indicate each individual value. Horizontal bars indicate mean value. Error bars indicate S.E.M. (* *p* < 0.05 vs. control animals; *n* = 7, respectively). (**K**) Representative photos of mitochondria (Mito) and LONP1 in CA1 neurons following SE. (**L**) Quantification of the mitochondrial length in CA1 neurons. Open circles indicate each individual value. Horizontal bars indicate mean value. Error bars indicate S.E.M. (*^,#^ *p* < 0.05 vs. control siRNA and LONP1 siRNA, respectively; *n* = 7, respectively).

In the present study, we found that CDDO-Me facilitated mitochondrial fission by increasing ERK1/2- and JNK-mediated DRP1-S616 phosphorylation. However, it was unclear whether these effects are relevant to the reduced LONP1 activity or the additional CDDO-Me actions. To directly elucidate the role of LONP1 in mitochondrial dynamics, we applied LONP1 siRNA prior to SE induction. In control animals, LONP1 knockdown effectively reduced LONP1 expression in the hippocampus (*p* < 0.05 vs. control siRNA; Figure 7A,B). Although LONP1 knockdown did not affect seizure susceptibility in response to pilocarpine (Figure 7C,D), it attenuated up-regulation of LONP1 expression induced by SE (*p* < 0.05 vs. control siRNA; Figure 7A,B). However, LONP1 siRNA exacerbated SE-induced neuronal damage in the CA1 neurons, hilus neurons and dentate granule cells, unlike CDDO-Me (*p* < 0.05 vs. control siRNA; Figure 7E,F). Co-treatment of CDDO-Me with LONP1 siRNA ameliorated only CA1 neuronal death, but not hilus neurons and dentate granule cells, following SE (*p* < 0.05 vs. LONP1 siRNA; Figure 7E,F). Furthermore, LONP1 siRNA did not influence the expression and phosphorylation of DRP1, ERK1/2, JNK and PP2B under physiological- and post-SE conditions (Figure 8A–J). LONP1 knockdown could not inhibit SE-induced mitochondrial elongation in CA1 neurons (Figure 8K,L). However, co-treatment of CDDO-Me with LONP1 siRNA abrogated mitochondrial elongation induced by SE (*p* < 0.05 vs. LONP1 siRNA; Figure 8K,L). These findings indicate that CDDO-Me-mediated mitochondrial fission may be independent of LONP1 activity, and that up-regulation of LONP1 may be an adaptive response to protect neurons from SE.

## 4. Discussion

Aberrant cell cycle entry in post-mitotic neurons leads to neuronal death in various neurological diseases [35,36]. Similarly, SE increases the expression of cell cycle regulatory molecules such as cyclin D1 and CDK4, and evokes programmed necrosis in CA1 neurons through dysfunction of mitochondrial fission [8,10,16,17,18]. Imbalance of mitochondrial fusion/fission rate evokes cell degeneration: Dysfunction of mitochondrial fission (aberrant mitochondrial elongation) results in improper segregation of mitochondria and a decrease in ATP levels, which abrogates mitochondrial transports in dendrites or axons, and subsequently induces ATP deficiency in peripheral sites. Impaired mitochondrial fission also inhibits respiratory function in mitochondria that triggers excessive ROS production. Therefore, dysregulation of mitochondrial fission leads to cell degeneration [37,38,39]. Excessive mitochondrial fission (impaired mitochondrial fusion) also results in cell death. This is because fragmented mitochondria are not able to produce ATP, which impairs the detoxification of excess ROS and extrusion of intracellular Ca^2+^, and in turn increases mitochondrial ROS and susceptibility to apoptosis [15,34,40,41]. Indeed, abnormal mitochondrial fission induces PV cell apoptosis following SE [10,25]. Since CDDO-Me inhibits cyclin D1 and induces cell cycle arrest [19,20], it is likely that CDDO-Me would affect aberrant mitochondrial dynamics induced by SE. In the present study, we found that CDDO-Me reduced mitochondrial length in CA1 neurons and PV cells without altering mitochondrial LONP1 expression in control animals. Furthermore, CDDO-Me effectively ameliorated SE-induced CA1 neuronal death, but not PV cell loss, accompanied by abrogating abnormal mitochondrial elongation. Since CDDO-Me induces mitochondrial fission in cancer cells [22,31,32] and WY14643 (an enhancer of mitochondrial fission) attenuates SE-induced CA1 neuronal death by rescuing aberrant mitochondrial elongation [8,25], our findings indicate that CDDO-Me-induced mitochondrial fission may have a selective neuroprotective effect against SE-induced CA1 necrosis, but not PV cell apoptosis.

In the present study, CDDO-Me increased DRP1-S616, but not S637, phosphorylation without changing the expression of other molecular components of mitochondrial dynamics, such as OPA1 and MFN1/2. Thus, CDDO-Me increased the DRP1-S616/S637 phosphorylation ratio. In our previous studies [8,16,17], SE decreased DRP1 expression and the DRP1-S616/S637 phosphorylation ratio, accompanied by increased mitochondrial length and sphere formation in CA1 neurons. Furthermore, WY 14643 increases DRP1-S616 phosphorylation and the DRP1-S616/S637 phosphorylation ratio [8]. Therefore, it is likely that the decreased DRP1-S616/S637 phosphorylation ratio may lead to aberrant mitochondrial elongation following SE. Consistent with these previous reports, the present study reveals that SE diminished the DRP1-S616/S637 phosphorylation ratio, concomitant with abnormal mitochondrial elongation, which were abolished by CDDO-Me. These findings indicate that CDDO-Me may facilitate mitochondrial DRP1 localization via the increased DRP1-S616/S637 phosphorylation ratio, although we could not confirm the subcellular localization of DRP1.

DRP1-S616 phosphorylation is regulated by the ERK1/2 and JNK signal pathways, which facilitate mitochondrial fission [27,29,30]. However, DRP1-S637 phosphorylation by PKA leads to detached DRP1 from mitochondria, thus inhibiting mitochondrial fission [26,28]. Indeed, DRP1-S637 dephosphorylation by PP2B accelerates mitochondrial fission [34]. Consistent with previous studies demonstrating that CDDO-Me regulates ERK1/2 [20,31] and JNK phosphorylation [32,33], the present data also reveal that CDDO-Me activated ERK1/2 and JNK under physiological conditions, and mitigated SE-induced reductions in ERK1/2 and JNK phosphorylation. However, CDDO-Me did not influence PKA and PP phosphorylation under physiological- and post-SE conditions. Since ERK1/2 inhibitor deteriorated SE-induced CA1 neuronal damage concomitant with mitochondrial elongation [18], our findings indicate that CDDO-Me may facilitate DRP1-mediated mitochondrial fission by activating ERK1/2 and JNK, which may attenuate CA1 neuronal degenerations following SE.

The present study also reveals that SE increased LONP1 expression in CA1 neurons as well as PV cells. LONP1 is one of the quality control proteins in the mitochondria supporting cell viability via the degradation of misfolded and damaged proteins under oxidative, hypoxic and endoplasmic reticulum-stress conditions [42,43]. Indeed, LONP1 expression is up-regulated under these stressful conditions [44,45]. Thus, LONP1 knockdown results in the disruption of mitochondrial function, reduced proliferation and the fragmented shape of the mitochondrial network [22,24,46]. With respect to these reports, it is likely that the up-regulation of LONP1 in PV cells and CA1 neurons may be an adaptive response to protect these neurons from SE. Considering this hypothesis and CDDO-Me as a LONP1 inhibitor [21,22], it is plausible that CDDO-Me would exacerbate degenerations of hilus interneurons and CA1 neurons by inhibiting LONP1 activity following SE. In the present study, however, CDDO-Me selectively alleviated CA1 neuronal damage without affecting the up-regulation of LONP1 expression induced by SE. In contrast, LONP1 knockdown aggravated neuronal loss of CA1 neurons. What makes these discrepancies in the effects of CDDO-Me and LONP1 siRNA on neuronal viability? In previous studies [21,22,24,46], CDDO-Me and LONP1 siRNA induced apoptosis of various cancer cells that have potent proliferative and differentiating abilities by inhibiting mitochondrial functionality. However, we applied CDDO-Me and LONP1 knockdown to post-mitotic neurons in the present study. Therefore, it is presumable that the differential proliferating ability of cancer cells and neurons would lead to the distinct effects of CDDO-Me and LONP1 siRNA on neuronal damage. Conversely, it is also considerable that LONP1 would not be a specific target of CDDO-Me. Indeed, the present data show that CDDO-Me affected ERK1/2 and JNK phosphorylation, while LONP1 siRNA did not influence the expression and phosphorylation of DRP1, ERK1/2, JNK, PKA and PPs under physiological- and post-SE conditions. In addition, co-treatment of CDDO-Me with LONP1 siRNA ameliorated only CA1 neuronal death, but not hilus neurons and dentate granule cells, following SE. Although LONP1 knockdown did not affect mitochondrial length in CA1 neurons, co-treatment of COOD-Me abrogated mitochondrial elongation induced by SE. Therefore, the present findings indicate that the neuroprotective effects of CDDO-Me may not be relevant to LONP1 inhibition. Since SE leads to programmed necrosis in CA1 neurons [8,16,17] and apoptosis in hilus interneurons, respectively [10], it cannot be excluded that CDDO-Me may selectively attenuate programmed necrosis rather than apoptosis. Taken together, the data from previous reports [8,25] and the present study identically suggest that the repair of dysfunction of mitochondrial fission may selectively rescue SE-induced CA1 neuronal death.

The heterogeneous vulnerability of hippocampal neurons in response to various insults has been reported. Briefly, dentate granule cells are remarkably resistant to various insults when compared to CA1 neurons or hilus interneurons [8,10,16,17,47,48,49]. Consistent with these previous studies, the present data show that SE induced a massive neuronal loss of hilus interneurons and CA1 neurons, although the degeneration of dentate granule cells was negligible. However, LONP1 knockdown provoked the massive degeneration of dentate granule cells, and aggravated loss of CA1 neurons and hilus interneurons. These findings indicate that LONP1 may be one of the important housekeeping molecules for neuronal viability, regardless of the heterogeneous vulnerability in response to harmful stimuli. Thus, it is likely that the regulation of LONP1 may be a useful therapeutic strategy for prevention of neurodegeneration. Future studies are needed to elucidate the role of LONP1 in other neurological diseases and the underlying regulatory mechanisms for its expression and activity.

## 5. Conclusions

To the best of our knowledge, the present data demonstrate, for the first time, the selective neuroprotective effects of CDDO-Me against SE. Briefly, CDDO-Me attenuated SE-induced CA1 neuronal death, but not hilus interneurons, by facilitating DRP1-mediated mitochondrial fission via ERK1/2 and JNK activation. Unlike CDDO-Me, LONP1 siRNA did not influence the expression and phosphorylation of DRP1, ERK1/2, JNK and PP2B under physiological- and post-SE conditions. In addition, LONP1 knockdown induced massive degeneration of dentate granule cells, and aggravated loss of CA1 neurons and hilus interneurons following SE. Co-treatment of CDDO-Me with LONP1 siRNA ameliorated CA1 neuronal death concomitant with abrogation of mitochondrial elongation induced by SE. Therefore, we provide an underlying mechanism of CDDO-Me for mitochondrial fission independent of LONP1 activity, and propose the availability of CDDO-Me for various neurological diseases relating to aberrant mitochondrial dynamics.

## Figures and Tables

**Table 1:** Primary antibodies used in the present study.

Antigen	Host	Manufacturer (Catalog Number)	Dilution Used
DRP1	Rabbit	Thermo (PA1-16987)	1:1000 (WB)
ERK1/2	Rabbit	Biorbyt (Orb160960)	1:2000 (WB)
JNK	Rabbit	Protein tech (10023-1-AP)	1:1000 (WB)
LONP1	Rabbit	Proteintech (15440-1-AP)	1:100 (IF) 1:1000 (WB)
MFN1	Rabbit	Proteintech (13798-1-AP)	1:1000 (WB)
MFN2	Rabbit	Proteintech (12186-1-AP)	1:1000 (WB)
Mitochondrial marker (Mitochondrial complex IV subunit 1, MTCO1)	Mouse	Abcam (#ab14705)	1:500 (IF)
OPA1	Rabbit	Abcam (ab42364)	1:1000 (WB)
pDRP1 S616	Rabbit	Cell Signaling (4494)	1:500 (WB)
pDRP1 S637	Rabbit	Cell Signaling (4867)	1:500 (WB)
pERK1/2	Rabbit	Bioss (bs-3330R)	1:1000 (WB)
PKA catalytic subunit	Rabbit	BioVision (3115-100)	1:1000 (WB)
PKA regulatory subunit	Rabbit	Santa Cruz (sc-909)	1:1000 (WB)
PP1	Rabbit	Abcam (ab52619)	1:5000 (WB)
PP2A	Rabbit	Cell Signaling (#2038)	1:5000 (WB)
PP2B	Rabbit	Millipore (07-068-I)	1:1000 (WB)
pPKA catalytic subunit	Rabbit	Assay Biotec (A0548)	1:1000 (WB)
pPKA regulatory subunit	Rabbit	GeneTex (GTX61061)	1:2500 (WB)
pPP1	Rabbit	Abcam (ab62334)	1:5000 (WB)
pPP2A	Rabbit	Sigma (SAB4503975)	1:1000 (WB)
pPP2B	Rabbit	Badrilla (A010-80)	1:1000 (WB)
PV	Goat	Swant (#PVG213)	1:10,000 (IF)
β-actin	Mouse	Sigma (A5316)	1:5000 (WB)
4-HNE	Rabbit	Alpha Diagnostic (# HNE11-S)	1:1000 (IF)

IF, Immunofluorescence; WB, Western blot.
